# A self-guided Internet-based intervention for individuals with chronic pain and depressive symptoms: study protocol of a randomized controlled trial

**DOI:** 10.1186/s13063-023-07440-8

**Published:** 2023-07-11

**Authors:** Swantje Borsutzky, Steffen Moritz, Birgit Hottenrott, Josefine Gehlenborg

**Affiliations:** Department of Psychiatry and Psychotherapy, University Medical Center Hamburg-Eppendorf, Martinistrasse 52, 20246 Hamburg, Germany

**Keywords:** Internet-based self-help, CBT, Chronic pain, Emotional problems, Online-intervention, Randomized controlled trial

## Abstract

**Background:**

Despite the existence of evidence-based therapy options for the treatment of chronic pain and comorbid depressive symptoms (e.g., CBT), many individuals remain untreated. Treatment gaps result from a lack of specialists, patient fear of stigmatization, or patient immobility. Internet-based self-help interventions could serve as an anonymous and flexible alternative treatment option. In a pilot study, chronic pain patients with comorbid depressive symptoms who used a generic Internet-based depression program showed a significant reduction in depressive symptoms (but not pain symptoms) compared to a waitlist control group. Based on these findings, we developed the low-threshold, anonymous, and cost-free Internet-based self-help intervention Lenio that is tailored to the specific needs of chronic pain patients with comorbid depressive symptoms. Lenio is accompanied by the smartphone application (app) COGITO designed to increase therapeutic success. With Lenio and COGITO addressing both chronic pain and depressive symptoms, the trial aims to increase treatment effects of online interventions for chronic pain patients by reducing both depressive symptoms and pain.

**Methods:**

The effectiveness of the Internet-based self-help intervention and accompanying smartphone app will be evaluated in a randomized controlled trial (RCT). A total of 300 participants will be randomized into an intervention group (Lenio/COGITO), an active control group (depression-focused smartphone app), or a waitlist control group. Assessments will be done at baseline, after an 8-week intervention period (post), and after 16 weeks (follow-up). The primary outcome is the post-assessment reduction in “pain impairment” (mean value of impairment in daily life, free time, and work) as assessed by the DSF (German pain questionnaire).

Secondary outcomes will include the reduction in depressive symptoms as well as in the severity of pain.

**Discussion:**

Lenio is one of the first Internet-based interventions to reduce chronic pain and depression that will be empirically evaluated. Internet-based interventions could offer a promising alternative to conventional face-to-face psychotherapy in the treatment of chronic pain patients. The primary objective of the current study is to add essential insight into the feasibility, effectiveness, and acceptance of Internet-based interventions for people with chronic pain and depressive symptoms.

**Trial registration:**

DRKS-ID DRKS00026722, Registered on October 6th, 2021.

## Introduction

### Background and rationale {6a}

#### The interaction of pain and emotional well-being

Pain is a psychophysiological warning sign that protects us from potential danger [36]. However, pain, especially when it manifests as a chronic condition, may become a profound problem for the affected individual. Worldwide, chronic pain is among the most common causes for seeking medical care, with a global prevalence of 15% to 45% and estimated economic costs in the US at $600 billion annually [49, 54]. Research findings highlight the negative effects of chronic pain on quality of life [10] in both private and professional life [16]. Considering these findings, it is not surprising that up to 75% of chronic pain patients experience comorbid depressive symptoms [32].

The relationship between chronic pain and depressive symptoms is likely bidirectional. On the one hand, depression can contribute to the multifactorial genesis of chronic pain [47], while on the other hand depressive symptoms can emerge as a result of impairments in psychosocial functioning and low quality of life due to chronic pain [44].

#### Psychotherapeutic treatment of pain

The gate control theory was the first model to emphasize the role of psychological factors on the experience of chronic pain, and its development initiated the integration of psychological strategies in the treatment of chronic pain [37]. Multiple studies found significant improvements in chronic pain and depression scores after psychotherapeutic interventions [22, 48, 55]. A meta-analysis by Veehof and colleagues [52] reported significant improvements in chronic pain intensity, and chronic pain interference at medium effect sizes after acceptance- and mindfulness-based interventions compared to wait-list, medical treatment-as-usual, as well as educational and support control groups. Although effective psychotherapeutic treatments like this are available, many individuals remain untreated. Psychological and social influences on the genesis and maintenance of chronic pain are often not recognized or addressed in standard pain treatment [38]. Furthermore, a significant amount of affected individuals experience (self-)stigmatization, which often accompanies depressive symptoms and is detrimental to patients’ motivation to seek psychotherapeutic treatment [11]. Moreover, immobility among pain patients may make traveling to healthcare institutions difficult [13, 18]. As a result, there is a need for low-threshold, easily accessible, and anonymous support services to help overcome the treatment gap of individuals with both chronic pain and depressive symptoms.

#### Internet-based psychological interventions

Internet-based psychological interventions present an alternative to conventional face-to-face psychotherapy in the treatment of chronic pain patients [8, 30, 48]. In recent years, many studies have replicated the effectiveness of Internet-based interventions for numerous mental disorders [8, 30, 48]. Firstly, Internet-based interventions are resource efficient and safe [21]. Secondly, due to flexibility in timing, intensity, and focus as well as anonymity and privacy, compliance with Internet-based programs is high [26, 25].

Internet-based interventions can be implemented as unguided (self-help) or guided treatment (individual support by a therapist, such as by telephone). According to a meta-analysis, guided Internet-based interventions have a similar effectiveness to conventional face-to-face therapy [8]. Most studies show greater effect sizes for guided than for unguided Internet-based interventions [46]. However, unguided Internet-based interventions are superior in terms of cost and resource efficiency as well as anonymity [25]. Their main drawback, adherence, may be counteracted by e-mail reminders and by presenting information in textual rather than non-textual format [1].

#### Internet-based psychological interventions for chronic pain

A recent systematic review of online interventions targeted at chronic pain found a small to moderate effect on pain intensity reduction (Hedges' *g* =  − 0.33) [6]. Programs that address both chronic pain and depressive symptoms are rare and randomized controlled trials (RCTs) examining their short- and long-term efficacy in large samples are scarce. To the best of our knowledge, there is no open-source Internet-based self-help program for the treatment of chronic pain and comorbid depressive symptoms.

Miegel and colleagues [39] evaluated an unguided Internet-based intervention named Novego for the treatment of depression in a sample of chronic pain patients. Significant reductions with small to moderate effect sizes in depressive symptoms in the Novego group were found compared to a wait-list control group (η_p_^2^ = 0.043). However, reductions in pain intensity compared to the wait-list control group were only observed for a small subgroup (e.g., pain localization in the upper back or hands). Hence, an Internet-based program specifically tailored to the individual needs of chronic pain patients with comorbid depressive symptoms, rather than depressive symptoms only, could augment these effects.

To provide a low-threshold, anonymous, and open-source Internet-based self-help intervention for chronic pain patients with comorbid depressive symptoms, we developed Lenio as part of the current research project. Lenio is a novel Internet-based self-help intervention addressing both chronic pain and depression. It is to be used in combination with a smartphone application (app) called COGITO (uke.de/cogito). Research has shown that the combination of Internet-based programs and smartphone apps that transfer psychotherapeutic strategies into everyday life may increase therapeutic success as well as adherence [51]. Previous RCTs of a pilot version of the COGITO app found significant reductions in self-reported depressive symptoms and an increase in self-esteem among those who used the app regularly compared to wait-list controls [5, 33].

### Objectives {7}

The aim of the current research project is to investigate the feasibility, effectiveness, and acceptance of the Internet-based self-help intervention Lenio in combination with the smartphone app COGITO in an RCT. As the primary objective, a significant reduction in pain impairment (mean value of impairment in daily life, leisure time, and work; primary outcome) as measured by the German Pain Questionnaire (German acronym: DSF) in the intervention group compared to a waitlist and an active control group is expected. Furthermore, we also assume that depressive symptoms, as well as pain-related cognitive biases (e.g., catastrophizing, fear-avoidance beliefs), will be significantly reduced in the intervention group compared to the two control groups. Hence, the treatment of a highly prevalent condition could be improved.

### Trial design {8}

The self-guided Internet-based intervention Lenio will be evaluated in the framework of a randomized controlled trial (RCT). Participants will be randomly allocated (ratio 1:1:1) to one of three conditions: (1) intervention group (direct access to the Internet-based intervention Lenio and the smartphone app COGITO), (2) waitlist control group, or (3) active control group (access to a transdiagnostic smartphone app named MCT & More).

The waitlist control will show the effect of the intervention against no treatment, while the active control will show the effect of the intervention versus an Internet-based intervention addressing psychological problems but not chronic pain {6b}.

## Methods: participants, interventions, and outcomes

### Study setting {9}

Data will be collected online at baseline, post intervention (8 weeks after baseline), and follow-up (16 weeks after baseline). The online surveys will be generated using the software Qualtrics®, which meets standards of data safety regulations. Both the waitlist control group and the active control group will receive access to the Internet-based programs Lenio and COGITO after completion of the follow-up assessment 16 weeks after baseline.

### Who will take informed consent? {26a}

At the online baseline survey, participants will be informed about the aims and the procedure of the study, inclusion and exclusion criteria, and their right to withdraw from study participation at any time. Subjects may request deletion of data already collected pending data evaluation.

### Data management {19}

All participants will be instructed to create an e-mail address without personally identifiable information. No additional personal data (e.g., names, addresses) will be collected. All e-mail addresses will be treated confidentially, will not be passed on to third parties outside of the research team, and will be deleted after the study’s end.

### Participant timeline {13}

After giving informed consent, sociodemographic and psychopathological data will be assessed. At the end of baseline, individuals will be randomized equally to one of the three conditions. Randomization will be conducted automatically in Qualtrics®, preventing influence by third parties {16a}. At post-intervention and follow-up, psychopathological data, subjective evaluation, and side effects will be examined. For participating in each of the three online surveys (baseline, post, follow-up), participants will receive €10 Amazon® vouchers (as much as €30 total) as an incentive.

Instructions after baseline will be delivered via e-mail. The intervention group will receive their login details for Lenio and COGITO. One week later, individuals in the intervention group who do not log into Lenio will receive a reminder, including an overview of the program. Adherence will be further enhanced by sending e-mails that include descriptions (including tips) of specific modules. The active control group will receive instructions and an MCT & More download link for Android and IOS. Both the active control and the waitlist control group will receive log-in details for Lenio after completion of the follow-up survey 16 weeks after baseline.

Links to the post and follow-up assessments will be sent by automatically triggered e-mails. If participants will have not participated in a particular survey, they will receive a maximum of two e-mail reminders at intervals of 1 week. (The first e-mail reminder will be automated. However, the second reminder will be a personal message from the principal study investigator) {18b} (Table 1).

**Table 1:**
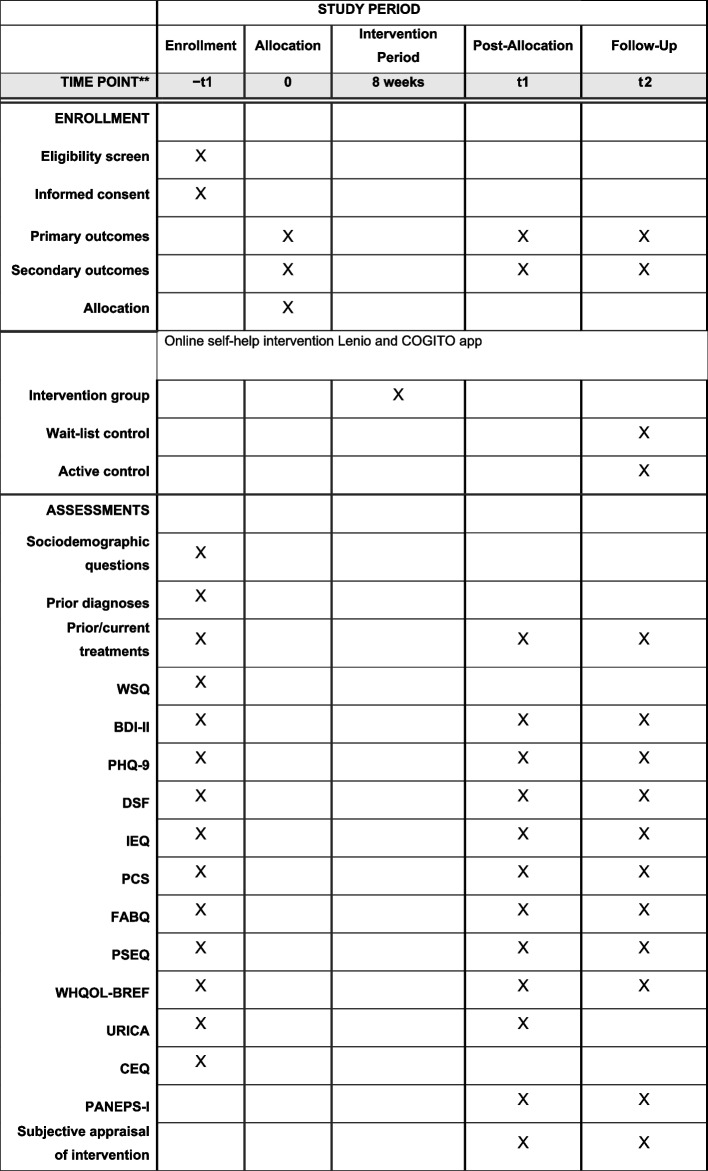
This table is based on the SPIRIT (Standard Protocol Items: Recommendation for Interventional Trials) timeline. BDI-II, Beck Depression Inventory-II; CEQ, Credibility/Expectancy Questionnaire; DSF, German Pain Questionnaire; FABQ, Fear-Avoidance Beliefs Questionnaire; PSEQ, Pain Self-Efficacy Questionnaire; IEQ, Injustice Experience Questionnaire; PCS, Pain Catastrophizing Scale; PHQ-9, Patient Health Questionnaire-9; URICA, University of Rhode Island Change Assessment Scale; WHQOL-BREF, World Health Organization Quality of Life abbreviated version; WSQ, Web Screening Questionnaire

### Sample size {14}

The sample size calculation was conducted using G*Power®. Given that Miegel and colleagues [39] examining the effect of an unguided Internet-based interventions for depression on chronic pain patients found small to moderate effects, a moderate effect in the current study can be expected [39]. For analysis of covariance, the software yielded a required sample size of 246 with an expected effect size of η_p_^2^ = 0.06 (moderate effect), an alpha of 0.05 (two-sided), and a power of 0.95. We expect a drop-out rate of 25%. Hence, we aim to recruit 300 participants in total, with 100 participants per condition.

### Recruitment {15}

Participants will be recruited in inpatient and outpatient clinics and institutions that will inform potential participants about the study and distribute the study flyers. Flyers will include study information and a QR code that leads directly to the baseline survey. Furthermore, study information and links will be distributed in Facebook groups regarding relevant topics (e.g., self-help groups for chronic pain). In addition, a Facebook/Instagram and Google Ads campaign has already been set up. The campaign targeted different groups of pain patients (e.g., pain caused by accidental injury). Further sources of recruitment are through self-help groups, Internet forums, websites and newsletters of health insurance companies/associations, and various social media platforms (Instagram, Reddit, Twitter, YouTube, LinkedIn), which is especially helpful in reaching people from the community and not only patients that are already in treatment.

### Eligibility criteria {10}

For inclusion in the RCT, participants must meet the following criteria:

- Presence of depressive symptoms (Beck Depression Inventory-II (BDI-II) score ≥ 14; Patient Health Questionnaire-9 (PHQ-9) score ≥ 10),
- Chronic pain symptoms (mean score pain intensity on the German Pain Questionnaire (DSF) ≥ 4),
- Aged 18 to 75 years,
- Informed consent,
- Sufficient knowledge of the German language,
- Willingness to participate in three anonymous online surveys,
- Willingness to use the Internet-based treatment program over a period of 8 weeks minimum, and
- Access to a computer/laptop and a smartphone.

The program has been developed for a heterogeneous target group of chronic pain patients with comorbid depressive symptoms. No formal diagnosis (of chronic pain and/or depressive symptoms) is needed as symptoms will be assessed by validated questionnaires at baseline. Effects of prior diagnoses as well as concurrent treatment programs will be analyzed in moderation analyses (see statistical analyses).

Exclusion criteria are a lifetime diagnosis of a schizophrenia spectrum disorder, bipolar disorder, substance abuse disorder, or acute suicidality (assessed by one item on suicidality on the Web Screening Questionnaire; WSQ).

Inclusion and exclusion criteria will be assessed at baseline. Excluded participants will be forwarded to an additional survey page providing the reason for exclusion and a contact e-mail address of the principal study investigator. In case of exclusion due to acute suicidality, participants will also be provided with phone numbers of crisis services.

## Interventions

### Intervention description {11a}

#### Lenio

The self-help Internet-intervention Lenio is based on principles of cognitive-behavior therapy (CBT) and its “third wave” properties (e.g., mindfulness-based and metacognitive techniques). Lenio includes a welcome module, an introduction module, and nine specific modules that all address chronic pain and emotional problems (see Table 2 for an overview of all modules). Some modules are more generic (e.g., social competence, self-worth), whereas other modules are more specific to chronic pain (e.g., modules on acceptance and commitment therapy [ACT], specific needs, and relapse prevention).

**Table 2:** Lenio modules

Module title	Module subunits	Description
Welcome		Motivate participants to work on psychological aspects of chronic pain; goal setting
Introduction	Introduction	Participants are introduced to various features of the program
	Things to know about pain	Information about chronic pain, pain management, emotional problems, and how these are all interrelated
	What can you expect from an Internet-based program?	Expectations and prejudices regarding Internet-based self-help programs are discussed
	Exercises to get you started	Introductory exercises introduce participants to Lenio
Basic needs	Basic needs	Psychological background of humans’ basic needs. The basic psychological needs are examined and put into the context of chronic pain management
Sleep	Introduction	Importance of sleep for mental and physical well-being
	Sleep disorders	Unhealthy sleep patterns are examined and explained through illustrations and examples
	Sleep hygiene	Improvement of participants’ sleep hygiene and thus improvement of overall health
	Cognitive biases	Cognitive biases related to sleep problems, strategies to modify dysfunctional thoughts
Attentional control and mindfulness	Attentional control	Integration of mindfulness exercises into everyday life
	Introduction to mindfulness	Concepts and background of mindfulness and its importance in the treatment of chronic pain are explained
	Mindfulness exercises for everyday life	Mindfulness techniques to reduce pain and improve participants’ mental health
	Mindfulness exercises with audio instructions	Various audio exercises make it easy to work on the implementation of these exercises
Modifying thoughts	Introduction	Dysfunctional thoughts and the consequences of these thoughts on pain and emotions
	Thinking distortions	Exercises to help turn dysfunctional thoughts into more realistic and helpful thoughts
	Dealing with thinking distortions/ABC scheme	Metacognitive elements
Acceptance and Commitment	Introduction	Introduction to the theory of acceptance and commitment therapy
	Acceptance	Theory of being accepting and the importance of acceptance
	Commitment	Theory of living a value-oriented life and minimizing energy spent fighting negative thoughts and feelings
	Take action!	Interactive elements and tools to encourage self-reflection and (re)orientation of one’s personal values
Social competence	Introduction to social skills	Examination of various social skills
	Representing one's own interests	Helpful advice on how to represent one’s interests
	Forming relationships	Helpful advice on how to form relationships
	Gaining sympathy	Helpful advice on how to develop communication skills
	Overcoming blockages/resistances	Demonstration of a set of strategies for cooperative and firm communication
Self-worth	Introduction	Relevance of self-worth to general mental health is emphasized
	Self-esteem	Participants are encouraged to identify their strengths
	Unfair comparisons	The effects of low self-esteem on chronic pain are addressed
Relapse prevention	My individual warning signals	Identifying one’s warning signals of relapse
	Introduction to self-determined relapse prevention	Helpful tools and resources to deal with relapse
	Emergency plan	Interactive worksheets with important personal information (e.g., pain medications, phone number of therapist) to use in an emergency
Incorporating positive activities	Introduction	The importance of positive activities for mental health and pain symptoms
	Stopping the downward spiral	Strategies to get out of passive withdrawal behavior and support to break out of a downward spiral
	Incorporating positive activities	Strategies to incorporate positive activities
	Goals	Goal setting

Modules are further divided into subunits. For example, the module *Sleep* is further divided into *Introduction*, *Sleeping problems*, *Sleep hygiene*, and *Cognitive distortions*. Participants are free to decide on the order in which they work on the modules. Once they decide on one module, they are advised to work through all its subunits in the given order. However, the division into subunits enables users to set priorities or to skip topics if they are already familiar with them. Moreover, participants do not have to work through one module in one session, as they can pause at any time and easily return to the last page visited, which will automatically be saved. Furthermore, we recommend they work through at least two modules per week. The time required to complete one module is 30 to 60 min on average.

When participants log into Lenio for the first time, an introductory video starts automatically that contains information on how to use Lenio. Subsequently, the *welcome module* starts, which was developed based on the theory of motivational interviewing. Participants start an interactive dialogue with an avatar. The aim is to motivate participants to work on the psychological aspects of their chronic pain. At the end of the *welcome module*, participants are introduced to five different avatars whose stories will be told in case vignettes throughout the program. All other modules include psychoeducational texts as well as interactive exercises and worksheets, graphics, videos, and audios. Participants are also provided with the possibility of communicating with a moderator via an internal messenger service. If participants have technical difficulties, the moderator will answer questions related to the program within three workdays. The moderator does not provide therapeutic guidance. Thus, Lenio is an unguided Internet-based intervention.

#### COGITO

The smartphone app COGITO can be accessed and downloaded via links to the Google Play Store (for Android users) and the App Store (for iOS users) that are provided in the desktop application Lenio. In the introduction module, participants are instructed on how to use the app. By sending daily push notifications with short exercises (reading time maximum 30 s) and providing elements of gamification (e.g., collecting medals for finished exercises), COGITO demonstrates high usability. Participants will be able to choose the time and frequency of push notifications. COGITO offers different packages of exercises (e.g. *Mood and Self-Esteem, Psychosis, Gambling Problems, OCD, Chronic Pain*). Participants will be instructed to activate the packages Chronic Pain and Mood & Self-Esteem. Most packages are deactivated by default but can be individually activated by the users. As with Lenio, COGITO exercises are based on CBT and third-wave techniques.

#### MCT & More

The active control group will receive access to the smartphone app MCT & More. As MCT & More is a pilot version of the COGITO app, its design and concept are similar to COGITO. MCT & More contains three packages (Mood, Metacognitive Training, and Gambling). However, the app does not contain pain-specific exercises. Participants in the active control group will be free to choose the packages they want to receive exercises from.

### Outcomes {12}

The primary outcome will be pain impairment (mean value of impairment in daily life, free time, work) as assessed by the DSF (German pain questionnaire). The DSF is a commonly used, reliable, and validated instrument for the assessment of chronic pain [9]. We decided on pain impairment as the main outcome as Lenio especially targets the psychological aspects of chronic pain. Hence, we expect a significant improvement in different aspects of life, including daily life, leasure time, and work. The secondary outcomes will include depressive symptoms as well as the severity of pain symptoms. Furthermore, subjective evaluation and possible side effects will be assessed.

#### Primary outcomes

##### The German Pain Questionnaire (DSF)

The DSF is a reliable and valid self-report questionnaire used to assess pain symptoms [9, 41]. It captures pain in a multidimensional manner. It assesses pain location (e.g., head coded as “0” if no headache is reported and coded with “1” if it is reported), subjective description of pain, onset and course of pain, and the presence and characteristics of pain attacks. In addition, participants are asked to rate pain intensity (current intensity, mean intensity, highest intensity in the last 4 weeks, and tolerable intensity after successful treatment) on a 10-point scale (“no pain” to “strongest pain imaginable”). The number of days of illness and the pain-related impairment of everyday life (0 “no impairment” to 10 “total impairment”) are also assessed. The DSF assesses previous treatments, the number of stays in rehabilitation clinics, physician visits, surgeries, and comorbid illnesses.

Items of the DSF addressing well-being, anxiety, and depression will not be included in the survey as these dimensions are assessed by other questionnaires (e.g., BDI-II, PHQ-9). Moreover, some items will be excluded because they are irrelevant to this study. For all measurement time points, the pain intensity will be calculated according to von Korff [53].

#### Secondary outcomes

##### Beck Depression Inventory-II (BDI-II)

The BDI-II [3] is a 21-item self-report questionnaire that assesses depressive symptoms over the past two weeks. Scores range from 0 to 63, with higher scores indicating higher levels of depression (0–8 = no depression, 9–13 = minimal depression, 14–19 = mild depression, 20–28 = moderate depression, and 29–63 = severe depression). Internal consistency is good, with Cronbach’s α of 0.89 [17]. In a sample of pain patients, comparable results were found [7, 43]. For all measuring points the BDI will be assessed. For our analysis, the sum score will be calculated.

##### Patient Health Questionnaire-9 depression module (PHQ-9)

The PHQ-9 [2, 29] is a self-report questionnaire measuring depressive symptom severity over the previous week. The PHQ-9 has high internal consistency (Cronbach’s α = 0.86–0.89, [29]. Its scores range from 0 to 27, with scores from 0 to 4 indicating minimal depression, 5–9 mild depression, 10–14 moderate depression, and 15–27 severe depression. For all measuring points, the sum score will be calculated.

##### Web Screening Questionnaire (WSQ)

The WSQ is a brief online self-report instrument that screens for common mental disorders such as affective disorders, alcohol abuse/dependence, general anxiety disorder, posttraumatic stress disorder, social phobia, panic disorder, agoraphobia, specific phobia, obsessive–compulsive disorder, and suicide risk. Sensitivity ranges between 0.72 and 1.00, and specificity ranges between 0.44 and 0.77 [14]. The WSQ will be assessed at baseline and for the analysis, the sum score as well as the cumulative values for all subscales of the WSQ will be calculated.

##### Injustice Experience Questionnaire (IEQ).

The IEQ is a valid and reliable tool for the assessment of perceived injustice in people suffering chronic pain, with a Cronbach’s α of 0.92 [50]. The IEQ consists of 12 items that assess thoughts of unfairness related to symptoms on a 5-point scale ranging from “never” to “all the time.” Perceived injustice is examined by the following elements: severity of loss consequent to injury, blame, sense of unfairness, and irreparability of loss. A score of 30 is the cutoff for a clinically relevant level of perceived injustice*.* For all three measuring points, the IEQ subscale “blame/injustice” and “severity/irreparability” as well as the sum score will be calculated.

##### Pain Catastrophizing Scale (PCS)

The PCS is a valid and reliable measure of catastrophizing. It assesses the thoughts and feelings of chronic pain patients. The questionnaire contains 13 items. Participants are asked which thoughts and feelings they experience during pain on a 5-point scale from “not at all” to “all the time.” Besides the total score, three subscales (rumination, magnification, and helplessness) are assessed. The PCS has demonstrated good internal consistency in the past with a Cronbach’s α of 0.87 [45]. For all three measuring points, the PCS total score as well as the subscores “helplessness,” “reinforcement,” and “worrying” will be calculated.

##### Fear-Avoidance Beliefs Questionnaire (FABQ)

The emergence of the biopsychosocial model of low back pain (LBP) led Waddell et al. [56] to develop the Fear-Avoidance Beliefs Questionnaire (FABQ, [56]). The FABQ assesses the patient’s fear-avoidance beliefs about physical activity and its contribution to lower back pain. It consists of 16 items that can be rated on a 7-point Likert scale (0 = “completely disagree” to 6 = “completely agree”). Test–retest reliability of the FABQ is excellent (ICC = 0.97, [27]). For the current study, we have adapted the FABQ to assess general chronic pain (not specifically lower back pain). For all three measuring points, the sum scores for “bodily activity” and “work load” will be calculated.

##### Pain Self-Efficacy Questionnaire (FESS)

To assess self-efficacy in our sample, we will use the German version of the Pain Self-Efficacy Questionnaire (FESS). According to Mangels and colleagues [34], the questionnaire is a valid instrument to measure therapeutic success in pain research. Specifically, it measures one’s conviction in one’s own ability to tackle activities despite suffering from pain. Additionally, it has high internal consistency, with a Cronbach’s α of 0.93. For all three measuring points, the sum scores of the PSE subscales “active coping” and “catastrophizing” will be assessed.

##### World Health Organization: Quality of Life abbreviated version (WHOQOL-BREF

The WHOQOL is a cross-cultural questionnaire assessing generic quality of life (QoL). QoL is defined as an individual’s perceptions of their position in life. QoL considers the context of culture and value systems, including personal goals, standards, expectations, and concerns. The questionnaire was developed by the WHOQOL Group of the World Health Organization. For the present study, we will use the global QoL item (“How would you assess your quality of life?”), with responses ranging from “very poor” (1) to “very good” (5). Despite its brevity, the internal consistency of the questionnaire was high (α = 0.90) in a sample of students [23]. For all measuring points, the global item will be assessed.

##### University of Rhode Island Change Assessment Scale (URICA)

The URICA is a self-report questionnaire developed to assess various stages of change in complex problem behavior [35].The URICA has demonstrated high internal consistency, with a Cronbach’s α of 0.77 to 0.82 and good reliability [15]. It predicts both treatment success as well as drop-out rates [35]. The present study will use the German version of the URICA (Fragebogen zur Erfassung der Veränderungsbereitschaf, FEVER, [19]). The FEVER captures three different phases of change: (1) pre-contemplation, i.e., lack of readiness to engage in an intervention or no readiness to change, (2) contemplation, i.e., readiness to engage in an intervention or to change something, and (3) action, i.e., the decision to change something and the use of active strategies to change. For the current study, a shorter version with only three items per phase (total of nine items) will be used. The FEVER/URICA will be assessed at baseline only. The subscales “precontemplation,” “contemplation,” “preparation,” “action,” and maintenance” will be calculated.

##### Credibility/Expectancy Questionnaire (CEQ)

For reasons of test economy, only one item of the CEQ [4, 12] will be used. The CEQ measures treatment expectancy (“What do you think at the present time about how successful the self-help program Lenio offered here will be in alleviating your complaints?”), which can be rated on a scale from 1 (“not successful at all”) to 9 (“very successful”). The CEQ item will be assessed at baseline only.

##### PANEPS

The Positive and Negative Effects of Psychotherapy Scale (PANEPS) is a self-report questionnaire that assesses positive effects and adverse events during the most recent session of psychotherapy [42]. The PANEPS consists of 29 items on four subscales: Positive Effects, Unethical Conduct, Malpractice, and Side Effects. Internal consistency is high for all subscales, with a Cronbach’s α of 0.72 to 0.92 [40]. In the current study, an adapted version (PANEPS-I) will be used that has not been validated yet (e.g., the wording of some items has been adapted). All four sub scores will be calculated for post and follow-up assessment.

##### Subjective appraisal of the program

The subjective appraisal of Lenio will be assessed using the German version of the Client Satisfactory Questionnaire (CSQ-8; German acronym is ZUF-8). According to the literature, the psychometrics of the CSQ-8 are high, with internal consistency ranging from Cronbach’s α = 0.87 to 0.93 [24, 28]. Satisfaction can be assessed on a 4-point rating scale (“excellent,” “good,” “less good,” “bad”). A high score indicates high satisfaction. The wording has been adapted by replacing “psychotherapy” with “self-help intervention Lenio.” Additional subjective data will be assessed by 12 further questions on the quality, utility, and applicability of Lenio in both open and closed response format (see Table 3).

**Table 3:** Subjective quality, utility, and application of Lenio

1. How regularly did you use the Internet-based self-help program Lenio?	◦ **Daily** **◦ 5–6 times per week** **◦ 3–4 times per week** **◦ 1–2 times per week** **◦ Less than once a week** **◦ Less than 5 times in the whole period** **◦ I have only used Lenio once**
2. Which topics were you able to complete in Lenio?	**◦ Introduction** **◦ Basic needs** **◦ Attentional control and mindfulness** **◦ Sleep** **◦ Self-worth** **◦ Modifying thoughts** **◦ Relapse prevention** **◦ Social competence** **◦ ACT** **◦ Incorporating positive activities**
3. I think Lenio is suitable for self-application	**◦ Applies** **◦ Rather applies** **◦ Rather not applicable** **◦ Not applicable** **◦ No answer possible**
4. I found Lenio to be helpful	**◦ Applies** **◦ Rather applies** **◦ Rather not applicable** **◦ Not applicable** **◦ No answer possible**
5. I think the instructions were written comprehensibly	**◦ Applies** **◦ Rather applies** **◦ Rather not applicable** **◦ Not applicable** **◦ No answer possible**
6. I was able to use Lenio regularly over the past weeks	**◦ Applies** **◦ Rather applies** **◦ Rather not applicable** **◦ Not applicable** **◦ No answer possible**
7. I had to push myself to use Lenio	**◦ Applies** **◦ Rather applies** **◦ Rather not applicable** **◦ Not applicable** **◦ No answer possible**
8. I feel that Lenio has reduced my chronic pain	**◦ Applies** **◦ Rather applies** **◦ Rather not applicable** **◦ Not applicable** **◦ No answer possible**
9. I feel that Lenio has reduced my emotional problems	**◦ Applies** **◦ Rather applies** **◦ Rather not applicable** **◦ Not applicable** **◦ No answer possible**
10. What would help you to better integrate Lenio into your everyday life?	
11.What did you like about Lenio?	
12. Do you have any suggestions for improvement?	

The answers to the seven closed questions will be assessed on a four-point Likert scale (“not applicable” to “completely true”). The open questions ask for positive and negative feedback on Lenio and will ask for improvement suggestions.

In addition, the participants will be asked to indicate how regularly Lenio was used during the intervention period. Subjective data will be assessed at post intervention and follow-up.

## Statistical methods

### Statistical methods for primary and secondary outcomes {20a}

All analyses will be conducted using IBM SPSS Statistics® 27. The data will be exported from Qualtrics® into SPSS® and initially stored on a secure server and later a password-protected computer. The security of data transmission via the Internet is also guaranteed by SSL encryption. Data will be analyzed using analysis of covariance (ANCOVAs), with between-group differences over time (pre-intervention to post-intervention) as the within-group factor and different conditions as the between-group factor. Baseline scores will serve as covariates, group allocation as the independent variable, and post- and follow-up scores of primary and secondary measures as dependent variables. Group differences at baseline will be assessed using independent t-tests for continuous variables. Partial eta square will be calculated as effect size.

### Interim analyses {21b}

For the current trial, an interim report has been written on 03/31/2022.

### Methods for additional analyses (e.g., subgroup analyses) {20b}

Explorative moderation analysis will be conducted using the SPSS® macro PROCESS [20] to identify potential moderators for treatment success (all types of baseline variables are being considered in this type of analysis). Furthermore, subjective evaluation of the intervention will be done descriptively by presenting the frequency of positive ratings as well as means and standard deviations.

### Methods in analysis to handle protocol non-adherence and any statistical methods to handle missing data {20c}

Analyses will be conducted for both the intention-to-treat (ITT) and the per-protocol (PP) sample. The PP sample will include only those participants who completed the post-assessment and used Lenio and COGITO at least once. Missing values in the ITT analyses will be estimated using the multiple imputation procedure.

### Plans to give access to the full protocol, participant-level data, and statistical code {31c}

Full protocol, dataset, and statistical codes will be provided upon request.

### Data monitoring and auditing {21a; 23}

An interim report was prepared for the DGUV, who are funding the project, in March 2022. First data was analyzed for this purpose.

### Ethical aspects and data safety

The study will be conducted in accordance with the Guidelines for Assuring Good Scientific Practice and the Principles and Responsibilities for the Conduct of Clinical Trials of the German Research Foundation (DFG). An ethics vote in support of the project has been obtained from the Local Psychological Ethics Committee of the Center of Psychosocial Medicine at the University Medical Center Hamburg-Eppendorf (LPEK-0078a). The study will be conducted in accordance with the Declaration of Helsinki. Any adverse events will be documented and reported {22}. Participants must sign informed consent prior to study participation, which informs them about the study procedure, data protection risks and compensation, and their right to withdraw from participation at any time without giving a reason. If they withdraw, all their data will be deleted.

The research project will be carried out in accordance with the EU General Data Protection Regulation. All other data will be stored on password-protected computers. Data collection will be conducted online using the survey software Qualtrics®, which guarantees high data protection standards such as end-to-end encryption. After completion of the study, the collected data will be archived for 10 years (e-mail addresses will be deleted after the end of the study). The clinical principal investigator (PI) will be responsible to carry out the study in accordance with the study protocol {5d}. The PI will ensure that everyone working in the research project is sufficiently qualified.

### Current study status

Trial start date: November 8th 2021 (first participant was enrolled).

Recruitment is completed.

## Discussion

Lenio is a novel Internet-based self-help intervention addressing symptoms of both chronic pain and depression. Whereas there is good evidence for the effectiveness of Internet-based interventions (guided and unguided) for depression and other mental disorders [8, 25, 30], programs targeting chronic pain and depressive symptoms are rare and often poorly validated, with a lack of data on long-term effects [6, 31]. RCTs with high methodological standards that examine both the short-term and long-term effects of Internet-based interventions for people with chronic pain and comorbid depression in large samples are scarce.

In the present study, we aim to evaluate the feasibility, effectiveness, and acceptance of Lenio in an RCT. To do so, we intend to reach out to individuals who either have not (yet) sought conventional face-to-face therapy or who are seeking complementary treatment, thus narrowing the existing treatment gap. The advantages of Internet-based interventions such as Lenio are their low cost, anonymity, flexibility, and low-threshold access. To the best of our knowledge, no Internet-based program exists that concurrently treats patients with both chronic pain and comorbid depressive symptoms. To offer complete anonymity for participants, we developed an unguided Internet-based intervention. Offering direct support might increase effectiveness but could also prevent patients from participating due to fear or self-stigmatization.

To increase adherence, we developed a smartphone app to be used in combination with Lenio. In addition, e-mail reminders will remind participants to access and use the program. Moreover, we integrated a welcome module containing motivational interviewing techniques to increase participants’ motivation to work on psychological aspects of their chronic pain. Within the welcome module, participants will receive an individualized recommendation of the modules most effective for them. Personal goals can be set and easily adjusted at any time.

One possibility for the future is that Lenio could be used as an after-care tool (e.g., after inpatient treatment) to prevent relapse. In addition, due to limited resources (e.g., too few specialists) patients currently have long wait times before they can start therapy. Internet-based interventions could be used to bridge this waiting time and provide patients with psychoeducational information and psychotherapeutic interventions. When they later start therapy, the time gained can be used to address other aspects (e.g., interpersonal problems, comorbidities) of treatment. Furthermore, participation in an Internet-based intervention could facilitate motivation to engage in face-to-face treatment and thus prepare patients for conventional face-to-face therapy. To shed light on the question of which baseline characteristics affect the outcome (e.g., if no add-on effects emerged when Lenio is used in addition to standard treatment), a moderation analysis will be calculated.

Currently, CBT interventions are most effective for patients with chronic pain and for depressed patients [22]. Lenio is based on CBT and its third-wave techniques. We expect to add to the understanding of the feasibility, effectiveness, and acceptance of Internet-based interventions for people with chronic pain and depressive symptoms. We expect that Lenio will significantly reduce chronic pain impairment as well as depressive symptoms when compared to the control groups (waitlist control group and active control group). In addition, we expect that the effects will be maintained long term as assessed by the follow-up measurement 4 months after the baseline assessment.

## Trial status

Protocol version 03/22/2023. Second revision.

Any future changes to the study protocol will be recorded in separate amendment. See Table 4 for a detailed SPIRIT 2013 Checklist.

**Table 4:** SPIRIT 2013 Checklist

Section/item	ItemNo	Description	Reported on page NO
**Administrative information**			
Title	1	Descriptive title identifying the study design, population, interventions, and, if applicable, trial acronym	1 (1)
Trial registration	2a	Trial identifier and registry name. If not yet registered, name of intended registry	2 (46)
	2b	All items from the World Health Organization Trial Registration Data Set	3
Protocol version	3	Date and version identifier	3 (table)
Funding	4	Sources and types of financial, material, and other support	3–4 (table)
Roles and responsibilities	5a	Names, affiliations, and roles of protocol contributors	1 (6) and 27 (524)
	5b	Name and contact information for the trial sponsor	3 (table)
	5c	Role of study sponsor and funders, if any, in study design; collection, management, analysis, and interpretation of data; writing of the report; and the decision to submit the report for publication, including whether they will have ultimate authority over any of these activities	3 (table)
	5d	Composition, roles, and responsibilities of the coordinating center, steering committee, endpoint adjudication committee, data management team, and other individuals or groups overseeing the trial, if applicable (see Item 21a for data monitoring committee)	23 (445)
**Introduction**			
Background and rationale	6a	Description of research question and justification for undertaking the trial, including a summary of relevant studies (published and unpublished) examining benefits and harms for each intervention	4 (57)
	6b	Explanation for choice of comparators	7 (144)
Objectives	7	Specific objectives or hypotheses	6 (128)
Trial design	8	Description of trial design including the type of trial (e.g., parallel-group, crossover, factorial, single group), allocation ratio, and framework (e.g., superiority, equivalence, noninferiority, exploratory)	6 (138)
**Methods: Participants, interventions, and outcomes**			
Study setting	9	Description of study settings (e.g., community clinic, academic hospital) and list of countries where data will be collected. Reference to where list of study sites can be obtained	7 (148)
Eligibility criteria	10	Inclusion and exclusion criteria for participants. If applicable, eligibility criteria for study centers and individuals who will perform the interventions (e.g., surgeons, psychotherapists)	10 (204)
Interventions	11a	Interventions for each group with sufficient detail to allow replication, including how and when they will be administered	11 (229)
	11b	Criteria for discontinuing or modifying allocated interventions for a given trial participant (e.g., drug dose change in response to harms, participant request, or improving/worsening disease)	7 (157) 23 (440)
	11c	Strategies to improve adherence to intervention protocols, and any procedures for monitoring adherence (e.g., drug tablet return, laboratory tests)	6 (122) 8 (171) 24 (465)
	11d	Relevant concomitant care and interventions that are permitted or prohibited during the trial	n/a
Outcomes	12	Primary, secondary, and other outcomes, including the specific measurement variable (e.g., systolic blood pressure), analysis metric (e.g., change from baseline, final value, time to event), method of aggregation (e.g., median, proportion), and time point for each outcome. Explanation of the clinical relevance of chosen efficacy and harm outcomes is strongly recommended	9 (table) 16 (277)
Participant timeline	13	Time schedule of enrolment, interventions (including any run-ins and washouts), assessments, and visits for participants. A schematic diagram is highly recommended (see Table 1)	7 (162) 8 (Table 1)
Sample size	14	Estimated number of participants needed to achieve study objectives and how it was determined, including clinical and statistical assumptions supporting any sample size calculations	10 (190)
Recruitment	15	Strategies for achieving adequate participant enrolment to reach target sample size	10 (193)
**Methods: Assignment of interventions (for controlled trials)**			
Allocation:			
Sequence generation	16a	Method of generating the allocation sequence (e.g., computer-generated random numbers), and list of any factors for stratification. To reduce the predictability of a random sequence, details of any planned restriction (e.g., blocking) should be provided in a separate document that is unavailable to those who enroll participants or assign interventions	7 (164)
Allocation concealment mechanism	16b	Mechanism of implementing the allocation sequence (e.g., central telephone; sequentially numbered, opaque, sealed envelopes), describing any steps to conceal the sequence until interventions are assigned	7 (164)
Implementation	16c	Who will generate the allocation sequence, who will enroll participants, and who will assign participants to interventions	7 (164)
Blinding (masking)	17a	Who will be blinded after assignment to interventions (e.g., trial participants, care providers, outcome assessors, data analysts), and how	n/a
	17b	If blinded, circumstances under which unblinding is permissible, and procedure for revealing a participant’s allocated intervention during the trial	n/a
**Methods: Data collection, management, and analysis**			
Data collection methods	18a	Plans for assessment and collection of outcome, baseline, and other trial data, including any related processes to promote data quality (e.g., duplicate measurements, training of assessors) and a description of study instruments (e.g., questionnaires, laboratory tests) along with their reliability and validity, if known. Reference to where data collection forms can be found, if not in the protocol	n/a see {12} for instruments 7 (150)
	18b	Plans to promote participant retention and complete follow-up, including list of any outcome data to be collected for participants who discontinue or deviate from intervention protocols	8 (176)
Data management	19	Plans for data entry, coding, security, and storage, including any related processes to promote data quality (e.g., double data entry; range checks for data values). Reference to where details of data management procedures can be found, if not in the protocol	7 (157) 22 (403)
Statistical methods	20a	Statistical methods for analyzing primary and secondary outcomes. Reference to where other details of the statistical analysis plan can be found, if not in the protocol	22 (399)
	20b	Methods for any additional analyses (e.g., subgroup and adjusted analyses)	22 (409)
	20c	Definition of analysis population relating to protocol non-adherence (e.g., as randomized analysis), and any statistical methods to handle missing data (e.g., multiple imputation)	22 (414)
**Methods: Monitoring**			
Data monitoring	21a	Composition of data monitoring committee (DMC); summary of its role and reporting structure; statement of whether it is independent from the sponsor and competing interests; and reference to where further details about its charter can be found, if not in the protocol. Alternatively, an explanation of why a DMC is not needed	23 (425)
	21b	Description of any interim analyses and stopping guidelines, including who will have access to these interim results and make the final decision to terminate the trial	22 (407)
Harms	22	Plans for collecting, assessing, reporting, and managing solicited and spontaneously reported adverse events and other unintended effects of trial interventions or trial conduct	23 (433)
Auditing	23	Frequency and procedures for auditing trial conduct, if any, and whether the process will be independent from investigators and the sponsor	23 (429)
**Ethics and dissemination**			
Research ethics approval	24	Plans for seeking research ethics committee/institutional review board (REC/IRB) approval	23
Protocol amendments	25	Plans for communicating important protocol modifications (e.g., changes to eligibility criteria, outcomes, analyses) to relevant parties (e.g., investigators, REC/IRBs, trial participants, trial registries, journals, regulators)	n/a
Consent or assent	26a	Who will obtain informed consent or assent from potential trial participants or authorized surrogates, and how (see Item 32)	23 (434)
	26b	Additional consent provisions for collection and use of participant data and biological specimens in ancillary studies, if applicable	n/a
Confidentiality	27	How personal information about potential and enrolled participants will be collected, shared, and maintained in order to protect confidentiality before, during, and after the trial	23 (428)
Declaration of interests	28	Financial and other competing interests for principal investigators for the overall trial and each study site	26 (518)
Access to data	29	Statement of who will have access to the final trial dataset, and disclosure of contractual agreements that limit such access for investigators	26 (516)
Ancillary and post-trial care	30	Provisions, if any, for ancillary and post-trial care, and for compensation to those who suffer harm from trial participation	n/a
Dissemination policy	31a	Plans for investigators and sponsor to communicate trial results to participants, healthcare professionals, the public, and other relevant groups (e.g., via publication, reporting in results databases, or other data sharing arrangements), including any publication restrictions	26 (521)
	31b	Authorship eligibility guidelines and any intended use of professional writers	27 (524)
	31c	Plans, if any, for granting public access to the full protocol, participant-level dataset, and statistical code	n/a
**Appendices**			
Informed consent materials	32	Model consent form and other related documentation given to participants and authorized surrogates	n/a
Biological specimens	33	Plans for collection, laboratory evaluation, and storage of biological specimens for genetic or molecular analysis in the current trial and for future use in ancillary studies, if applicable	n/a

## Data Availability

Data is available upon request. Data will be communicated via publications on peer-reviewed journals and poster presentations or oral presentations at conferences.

## Abbreviations

BDI-II: Beck Depression Inventory-II
CEQ: Credibility/Expectancy Questionnaire
DSF: German Pain Questoinnaire
FABQ: Fear-Avoidance Beliefs Questionnaire
PSEQ: Pain Self-Efficacy Questionnaire
IEQ: Injustice Experience Quesstionnaire
PCS: Pain Catastrophizing Scale
CBT: Cognitive-behavioral therapy
RCT: Randomized controlled trial
PHQ-9: Patient Health Questionnaire-9
URICA: University of Rhode Island Change Assessment
WHQOL-BREF: World Health Organization Quality of Life abbreviated version
WSQ: Web Screening Questionnaire
